# Using Extended Technology Acceptance Model to Assess the Adopt Intention of a Proposed IoT-Based Health Management Tool

**DOI:** 10.3390/s22166092

**Published:** 2022-08-15

**Authors:** Dewen Liu, Qi Li, Shenghao Han

**Affiliations:** 1 School of Management, Nanjing University of Posts and Telecommunications, Nanjing 210003, China; 2 School of Economic Management, Tongji University, Shanghai 200092, China; 3 College of Business, Shanghai University of Finance and Economics, Shanghai 200433, China

**Keywords:** Internet of Things, digital health, optical sensor, technology acceptance model, MOA theory

## Abstract

Advancements in IoT technology contribute to the digital progress of health science. This paper proposes a cloud-centric IoT-based health management framework and develops a system prototype that integrates sensors and digital technology. The IoT-based health management tool can collect real-time health data and transmit it to the cloud, thus transforming the signals of various sensors into shared content that users can understand. This study explores whether individuals in need tend to use the proposed IoT-based technology for health management, which may lead to the new development of digital healthcare in the direction of sensors. The novelty of this research lies in extending the research perspective of sensors from the technical level to the user level and explores how individuals understand and adopt sensors based on innovatively applying the IoT to health management systems. By organically combining TAM with MOA theory, we propose a comprehensive model to explain why individuals develop perceptions of usefulness, ease of use, and risk regarding systems based on factors related to motivation, opportunity, and ability. Structural equation modeling was used to analyze the online survey data collected from respondents. The results showed that perceived usefulness and ease of use positively impacted adoption intention, Perceived ease of use positively affected perceived usefulness. Perceived risk had a negative impact on adoption intention. Readiness was only positively related to perceived usefulness, while external benefits were positively related to perceived ease of use and negatively related to perceived risk. Facilitative conditions were positively correlated with perceived ease of use and negatively correlated with perceived risk. Technical efficacy was positively related to perceived ease of use and perceived usefulness. Overall, the research model revealed the cognitive mechanism that affects the intention of individuals to use the system combining sensors and the IoT and guides the digital transformation of health science.

## 1. Introduction

In sensing technology, rapid advancements have been observed in detecting and improving individuals’ health status [1]. For example, pulse oximetry is a painless, non-invasive method of measuring oxygen saturation in a person’s blood, providing essential health data for users to monitor their degree of blood oxygen saturation. When people were “blocked” at home during the COVID-19 epidemic, in-house light sensors helped monitor individuals’ behavioral patterns and remotely judged their vital signs.

In the past years, there have been tremendous advancements in different specializations of health science, such as remote diagnosis and treatment, medical care, and health monitoring which has led to humans finding newer and better ways to tackle health problems and prevent emerging situations. Multiple reports from IDTechEx [2,3], for example, show that the global market for electronic skin patches in wearable medical devices will exceed $10 billion in revenue in 2021 and is expected to exceed $30 billion by 2031, The market for medical piezoelectric sensing systems is expected to reach $1.04 billion by 2029. In addition, the global market for artificial intelligence image-based medical diagnostics will exceed $3 billion by 2030, and the market for flexible medical electronics will exceed $8.3 billion in the same period. However, although sensing technology can significantly improve people’s health, many people are skeptical of its application [4]. Especially regarding optical biosensors, which many discommenders argue would expose people’s personal privacy [5,6].

More commercially available sensing devices combined with the Internet of Things (IoT) help people share physiological and biochemical information to get more benefits through transmitting and analyzing these data. With the rapid adoption of IoT technology for various industries, it is predicted that by 2026, the IoT market will be valued at USD 1102.6 billion [7]. IoT-based applications have attracted voluminous academic attention [8], and researchers have been exploring the possibility of using IoT for every possible scenario, including health management.

With the combination of IoT and sensing technology, it is possible and inexpensive to implement a regulatory health management system and provide sound warnings to the users’ family members at an early stage of any emergency or provide practical suggestions for users to improve their health status. This combination of health management and application has recently gained the attention of many technology giants. For example, Apple has added blood oxygen sensors to the Apple Watch to monitor the user’s blood oxygen levels and to suggest adaptive adjustments that should be made.

In the information system (IS) research field, scholars also recently discovered the trend of combining health management and digital technologies. For example, Ref. [9] designed an approach to diagnose the on-time and future panic health state of people at fog layer, indicating a good predictive effect. Ref [10] developed a telemonitoring program via cardiac implantable electronic devices (CIEDs) in conducting Cardiac rehabilitation and secondary prevention. One study further argued that this kind of health management tool consists of new technical issues (e.g., privacy, literacy, accessibility) [11] and the willingness of individuals to accept such technological products determine the success or failure of the products [12].

**Figure 1:**
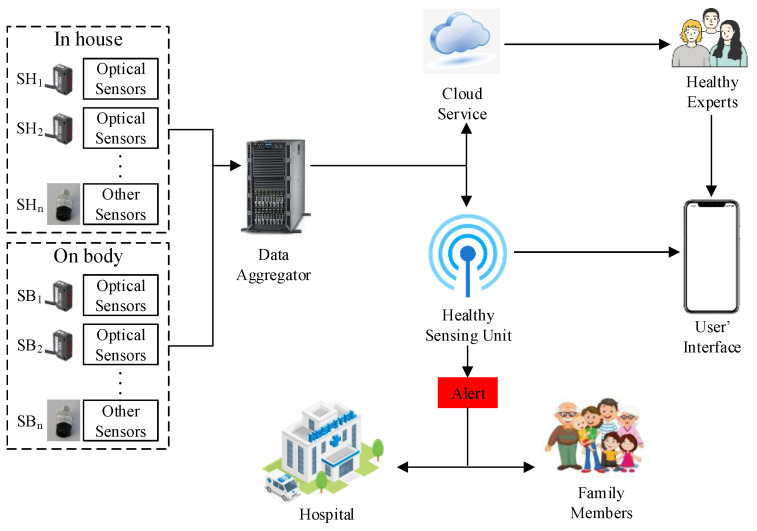
Proposed IoT-based framework for health management.

In this study, we propose a cloud-centric IoT-based health management framework prototype, as shown in Figure 1. This framework allows for the automatic reporting of health emergency to hospitals and users’ contacts (e.g., family members) without human intervention within a few seconds when related sensors detect the abnormal data. The users’ data is regularly uploaded to the cloud, and health experts analyze the data to make health recommendations for the users. Users can also check their health data and health status in real-time on their mobile devices. The framework also has provisions for continuous reporting of detailed information, thereby allowing health experts to effectively plan future diets or exercise dynamically according to the variation trend of physical signs. The foundation of this management system is optical sensors, which are responsible for monitoring basic indicators through forming images. Users can check real-time data in the interface of the mobile devices and control all functions. A diabetic patient, for example, is a user of this system. The user’s data is captured by sensors and transmitted to the sensing unit. The user can view various health data, including blood glucose, blood oxygen and pressure status, on the mobile interface of Applications. The optical sensors in the house also capture the user’s activity and remind him or her to exercise or take medication on time. The user can also turn these sensors on or off on the phone. At the same time, the user’s data is also transmitted to the cloud, and sent to the appropriate health experts. They analyze the user’s health data to find out the changes in the user’s physical status, such as decreasing blood sugar level. This leads to recommendations for appropriate health interventions, such as reducing the intake of bran foods. The user can also choose to turn off these health information pushes. However, since such IoT-based systems are not reportedly deployed, most individuals and health experts are unaware of the benefits of systems based on such frameworks. Therefore, it is imperative that individuals become acquainted with this innovative technology to enable their adoption. This paper developed a prototype of the system to inform the individuals who may need it about the proposed system. The prototype comprises the optical biosensors used by individuals in daily life and other related processing sensors. These details are dynamically fetched from a cloud database.

Various sensors, such as blood oxygen, motion tracking, image, and position sensors, are embedded in the framework. Some of the sensors are placed in the user’s living house (e.g., motion tracking sensors). Other sensors are wearable ones. For example, Ma et al. (2022) found that the correlation between strain and optical signals generated by surface wrinkle establishment demonstrates a novel design strategy for wearable optical sensors based on the superb strain sensitivity of the double-layer wrinkle system to surface wrinkles [13]. These sensors collect and provide real-time data to a data aggregator that amalgamates them into a data package with a panel form. The package is transmitted to the cloud and the sensing unit simultaneously. In the cloud, the data are in an online “storage”, containing other users’ information. This process is conducted for each set of sensor values, and the cloud could depict the normal numerical values of each indicator through constant deep learning and comparing. All data packages are stored in the right places under the supervision of regulations and laws.

The healthy sensing unit is also embedded as a customized service on the cloud, which turns the raw data into apprehensible content. The unit uses extensive data analysis to determine if the user is in a normal state of health. Suppose the unit detects that the user is in an unhealthy state (e.g., a body index that is far from standard), the unit would like to initiate an alert module and send the user’s health information to relevant parties, such as the contacts (e.g., family members) and hospitals that the user has set up. All associated parties that receive the message can initiate an emergency solution in the first instance to help the user get back to a healthy state.

## 2. Literature Review

### 2.1. IoT-Based Management

The Internet of Things (IoT) describes physical objects with sensors that connect and exchange data with other devices over the Internet or other communications networks. The emergence of IoT has changed the traditional approaches to managing various sensors and opened new perspectives for digital health that delivers useful and real-time information [14]. Many studies have examined sensors and IoT together and concluded that IoT-based sensor collaboration systems could often be more effective. For example, based on the need for physiological parameter monitoring during the rehabilitation training process, the system constructed with IoT can closely monitor changes in vital signs of target users while providing real-time monitoring and feedback [15]. In addition, IoT technology can help detect minimal environmental changes [16], leading related parties to react to these changes swiftly.

Especially in health management, IoT is a promising solution for providing continuous and holistic detections and supporting clinical decision-making [17]. For the smart home, a networking system would be able to detect possible chronic disease of family members [18]. The combined system with smartphone and wearable sensors could also be used to prevent individuals’ anxiety, obesity, and disorders [19]. In our proposed framework, IoT connects various house and body sensors. By collecting real-time information for improving and tracking the images of users and objects, processing and analyzing the data, and providing specific suggestions, IoT can stimulate and magnify the usefulness of sensors. Users can obtain real-time information on the condition of their comprehensive health indicators on their devices (e.g., smartphones, personal computers, and laptops) to improve their health status and improve their quality of life. Other parties could also observe the images of the focal user.

### 2.2. Technology Acceptance in IS Field

The acceptance of technology related products is the core in information system research. Studies found that older adults resist adoption of such technological products, leading to the emergence of an age-based digital divide [20,21]. Ref. [21] proposed an integrated framework that combined protection motivation theory and social cognitive theory and this model was used to explore the mobile health adoption intention among US and Ireland citizens. The authors found that seniors’ perceived inability to adopt stemming stemmed from mistrust, high risk perceptions, and a strong desire for privacy. Ref. [22] conducted a meta-analysis to develop a synthetic framework regarding the adoption of mobile health services and found that perceived usefulness, perceived ease of use, perceived vulnerability and perceived severity significantly influenced individual attitude. Similar results could be observed in Ref. [23], which found that usefulness, convenience and monetary value of mobile health positively influenced adoption intention. Ref. [24] used a UTAUT2-based model to evaluate individuals’ adoption through Partial least squares structural equation modeling (PLS-SEM) and the fuzzy-set qualitative comparative analysis (fsQCA) approach and proved the possible combined conditions (e.g., performance expectancy, habit) in cultivating individuals’ willingness to adopt the health system. Based on trust and anxiety perspectives, Ref. [25] confirmed that affective and cognitive trust enhanced elderly users’ continuance intention and health anxiety moderated the relationship between trust and intention. The main barriers affecting individual adoption of mobile health come from technology, individual and system, including security/privacy concerns, knowledge and economic factors [26].

Scholars in the IS field have used different theories and different models to study the willingness of different groups of people to adopt or accept mobile health. The core variables (e.g., perceived usefulness, perceived ease of use) included in these studies are still derived from the TAM model. As Ref. [27] suggested, the model with PU and PEU accounted for 61% and 72% of the variance in predicting individuals’ adoption of technology products in the limited and expanded use groups, respectively. Compared to other contest models, the TAM model showed considerable power in explaining individuals’ intentions towards the proposed technical system [20].

### 2.3. TAM Model

Technology Acceptance Model (TAM) was put forward by Ref. [28], based on the Theory of Reasoned Action, which explains and predicts individuals’ adoption or acceptance of computer information systems or information technologies. This model puts forward two main determinants: perceived usefulness and perceived ease of use. Perceived usefulness refers to the degree to which users think using a specific system can improve their work efficiency or performance, and perceived ease of use refers to the degree to which users think the system is easy to use [28]. Meanwhile, perceived ease of use strengthens perceived usefulness in specific situations [20]. Due to the simplicity, ease of operation, and strong pertinence of the model, TAM theory has been applied in many technical fields, and with a change of research objects, different extended versions of TAM have been extended [29,30]. The perceived risk theory is often integrated with TAM to study consumer behavior. The concept of perceived risk was first extended from psychology by Ref. [31] and advocated by many studies [32]. Uncertainty about the outcome implicit in an individual’s decision was defined as risk.

Recent pieces of evidence show that information and communication technology (ICT) and the Internet of Things (IoT) have brought significant changes to the traditional environment of healthcare services [33,34]. However, it is a helpful health management tool only when people start to use it. Therefore, the overall attitude of end-users towards accepting digital health management tools plays an important role. In current studies, it was found that although TAM has been widely used in various fields, there are few studies on users’ adoption behavior in the digital healthcare environment, and most of them are from the single factor level [35]. Therefore, this study uses TAM as the main theoretical framework to develop a research model to evaluate the adoption intention of IoT-based health management tools. Individuals are in the stage of understanding and exploring IoT-based health management tools and are unfamiliar with its related operating mechanisms and systems. The information in the system is scattered, and the amount of information is extensive, leading to risk perception. Therefore, this study considers the impact of perceived risk, perceived usefulness, and perceived ease of use, and comprehensively explores the main facilitators and barriers to users’ adoption intentions of IoT-based health management tools.

### 2.4. MOA Theory

As a theoretical underpinning, motivation-opportunity-ability (MOA) theory underscores the motivation, opportunity, and ability to co-determine individuals’ desire to become involved in a particular behavior [36]. If all three factors are satisfied simultaneously, the odds of an individual performing a behavior are maximized. In the MOA framework, motivation pertains to the readiness, interest, and other related incentives to conduct information processing toward a particular behavior. The concept of opportunity is the degree to which a situation is conducive to achieving the desired outcome, representing the environmental stimulus that enables action. Ability refers to individuals’ skills or competency in “turning” an action into a natural action [37]. The MOA framework is based on theoretical discussions among behavioral scientists who believe behavior results from combining these three factors [38].

The MOA model has been used in varied research into acceptance of technological products, such as media use [39] and sustainable purchase [40]. Some studies have also combined this theory with other theoretical frameworks to get a deeper insight into individuals’ behaviors. For example, Ref. [41] used MOA factors as essential moderators in the relationship between charismatic leaders and service-sales ambidexterity. Ref. [42] integrated planned behavior theory (PBT) with MOA theory in entrepreneurial behavior research and found the performance and predictive power of the synthetical model compared to the individual models. Similar results were also observed in energy-saving behaviors [43] and label use [44], by the combination of MOA theory with other behavioral theories or models (e.g., norm activation model).

In this study, we argue that the TAM model alone does not explain the behavioral intentions of individuals toward the proposed system as antecedent factors of individuals’ perceptions were underappreciated in the original TAM model. In our proposed IoT-based framework, combining motivation (readiness and external benefits), opportunity (facilitating conditions) and ability (technical efficacy) with the TAM model can explain why individuals develop perceptions of usefulness, ease of use, and risk toward the system, which then influences their adopt intention. In this paper, readiness, as an intrinsic motivation, is defined as the degree to which users are ready to adopt and use the proposed system to achieve specific goals [45], which can positively influence users’ perception of new technologies and systems [46]. External benefits, as an external motivation, refer to the motivation and benefits beyond the system–user interaction itself [47], which is the concentrated embodiment of the extensive benefits and functional upgrading of the IoT-based health management framework. Facilitating conditions, as environmental opportunities, are defined as the objective factors in the environment recognized by users that make the adoption and use of the proposed system easy to complete [48], such as a user guide and concise and efficient interactive interface, which can improve the degree to which individuals believe that the infrastructure exists to support the system [48,49]; Technical efficacy refers to the confidence and necessary skills that users have to achieve specific goals by using the proposed system [50], which can significantly affect users’ understanding and adoption of new health management tools. To sum up, this paper holds that users’ perceptions of usefulness, ease of use, and risk toward the proposed IoT-based framework for health management are influenced by users’ willingness to prepare for the new technology framework (motivation), their own efficacy (abilities) and environmental factors (opportunities).

## 3. Hypotheses Development

### 3.1. Perceived Usefulness

Perceived usefulness refers to the degree to which users believe adopting a technology (platform/software) can increase their performance [51]. In the research of online services [52], mobile services [53,54], and the functions of new technologies/systems [55,56], perceived usefulness is mostly demonstrated and measured by the function and service upgrade degree and relative advantages of new technologies/systems, and there is a perfect measurement scale for reference. Specific to digital health management, perceived usefulness refers to the convenience brought by patient users who adopt and use the IoT-based health management tools to improve their quality of life. Compared with the traditional offline medical examination and other health management modes, the IoT-based health management tools can improve the transmission speed of medical information and provide timely health care suggestions for users. Users do not need to go through the long offline medical treatment process, such as queuing, registration, and seeing a doctor, and can, thereby, improve the efficiency and convenience of their lives. At the same time, detailed and continuous health reports and data visualization services can help patient users to see the general situation of health information and the relationship between data more intuitively. Therefore, when patients perceive the value in adopting IoT-based health management tools, the easier it is for them to adopt and use them. In addition, several studies have also confirmed the positive influence of perceived usefulness on adoption intention [57,58]. Therefore, we assume the following:

**Hypothesis** **1** **(H1).** *PU positively affects AI.*

### 3.2. Perceived Ease of Use

Perceived ease of use is the perception of the ease of using new technologies [28]. In the previous literature, perceived ease of use is mostly reflected by newbie friendliness and operation convenience of new technologies/systems, and there is a perfect measurement scale for reference [59,60]. In digital health management, perceived ease of use is defined as the ease with which patient users adopt and use the IoT-based health management tools [51,61]. For example, can patient users quickly realize a series of operations, such as real-time uploading and checking of their health data and health status, online diagnosis and treatment consultation, or disease information inquiry on the IoT-based health management tools, or is it easy for patient users to learn and master these operations? TAM asserts that the perception of ease of use broadly explains people’s perception of usefulness and their attitude towards using a specific system [62]. If all other factors remain the same, an easier-to-use system will improve the user’s productivity or performance [63,64]. When users do not have the necessary skills and confidence or do not know a specific system, giving full play to the system’s advantages is not easy.

On the contrary, when users can easily understand and use the system, the system’s functions will be effectively coordinated to serve users and respond to users’ needs in a targeted manner. Most users will now change their views on its usefulness [34]. Therefore, perceived usefulness should be increased by adding current functions that are easier to use in the system [65]. 

Meanwhile, if it is not easy to use the IoT-based health management tools, and users have to spend more time and energy on this particular system to learn how to use it, they may not like to use it or may give up using it. Therefore, the more concise and understandable the IoT-based health management tools are, the higher the fluency of operation, the better the user’s experience of the new system, and the stronger the intention to adopt and use it. In addition, several studies have confirmed the positive influence of perceived ease of use on adopt intention [66,67]. Therefore, we assume the following:

**Hypothesis** **2** **(H2).** *PEU positively affects PU.*

**Hypothesis** **3** **(H3).** *PEU positively affects AI.*

### 3.3. Perceived Risk

Perceived risk refers to the psychological danger that people feel because of the uncertain outcome of their own choices, including fear, hesitation and even some negative emotions [68]. This concept now has been widely used in research on the adoption of various new technologies [69,70], with a perfect measurement scale for reference. In the research of this paper, perceived risk refers to the user’s estimation of the severity of adverse consequences of digital health management system and the uncertainty and unfamiliarity of its safe and stable operation. Although the emergence and application of digital health management has brought much convenience to the majority of patients, it is, however, precisely because this process is carried out through the cloud online that there is perceived risk. In the process of adopting and using the IoT-based health management tools, there may be the possibility that private information will be leaked or illegally stolen, tampered with and used. This perceived risk reduces the user’s evaluation of the system, the degree to which the system improves the user’s quality of life, and the perception of the usefulness of the new system. 

Furthermore, if users think that their information is not safely protected in digital health management, the adoption of new technical solutions is reduced [34,70]. At the same time, when it comes to health care, people aremore cautious, lack a sense of adventure and innovation, have poor cognition and acceptance of new things, and are more inclined to choose traditional methods to avoid risks when the results are uncertain. In addition, many studies have analyzed that perceived risk leads to the expectation of destructive consequences, which hurts the adopt intention [71,72]. Therefore, we assume the following:

**Hypothesis** **4** **(H4).** *PR negatively affects PU.*

**Hypothesis** **5** **(H5).** *PR negatively affects AI.*

### 3.4. Motivation

Motivation refers to related incentives that urge someone to conduct a behavior, consisting of intrinsic and external motivation [73]. Intrinsic motivation is defined as conducting a behavior for its inherent satisfactions rather than for some separable consequences, and external motivation means that the reasons for conducting a behavior come from a source outside oneself [74]. In our framework, we argue that readiness and external benefits can reflect intrinsic and external motivation towards the proposed system.

Readiness (RS) is the state of readiness before a user performs a certain behavior [52]. Research has shown that when an individual has a solid internal tension to perform a behavior, the more relevant experience the individual has and the better the understanding of the process and role of the behavior, the more prepared the individual is to perform the behavior. That is, the more relevant experience he or she has, and the better he or she understands the process and role of performing the behavior so the more muscular the individual’s internal tension to perform a behavior is [75]. For a sensor- and IoT-based system, readiness allows users to have a better understanding of the “script” and the role they should play, to know when and how to participate in the process, and, therefore, to have a higher level of perceived usefulness and perceived ease of use to the system, and a lower level of perceived risk.

External benefits (EB) represent an individual’s benefits that have nothing to do with intrinsic or core benefits [47,76]. An individual extrinsically motivated to attain the focal object aims at utilitarian rewards, seeking assistance, or maintaining a sense of it being worthwhile [77,78]. The premise of an individual’s use of a technology system also depends on how much external utility (e.g., external benefits) they expect to derive from that technology system. Individuals also tend to develop positive perceptions of technology systems when they provide more external utility. Thus, the following hypotheses are put forward:

**Hypothesis** **6-1/2/3** **(H6-1/2/3).** *RS significantly affects PU/PEU/PR.*

**Hypothesis** **7-1/2/3** **(H7-1/2/3).** *EB significantly affects PU/PEU/PR.*

### 3.5. Opportunity

The proposed system is intended to be mainly used by individuals who need it (e.g., patients) and can relate the usefulness of the proposed system in executing their daily health monitoring activities. In our framework, the opportunity provides a “window” for users to approach the technological system. We use facilitating conditions to measure the individuals’ opportunity factors. Previous studies have confirmed the significant relationship between facilitating conditions and usage behavior [79].

Based on literature perspectives, we assume that facilitating conditions (FCs) are important variables that influence individuals’ perceptions [80]. In the context of the technological system, facility conditions include guidance on how to use it, the necessary knowledge, and the complexity of the expressed processing steps [81], helping the individuals to access the system with low effort. Thus, a positive correlation exists between facilitating conditions and perceptions of the proposed system, so the facilitating conditions are assumed to be a vital variable that affects both perceived usefulness, perceived ease of use, and perceived risk. The following hypotheses were accordingly derived.

**Hypothesis** **8-1/2/3** **(H8-1/2/3).** *FC significantly affects PU/PEU/PR.*

### 3.6. Ability

We use technical efficacy (TE) to measure individual ability to approach or use the proposed system. As new technologies are introduced in any domain, users often lack confidence. Accordingly, current studies have confirmed the positive effect of technological efficacy on innovative product usage as an important factor [82]. Since the proposed system in this study is a health management tool for obtaining essential data to enable effective detection in case of emergencies, we also considered technical efficacy in the proposed system because it could have a direct effect on individuals’ perceptions of the system, including perceived usefulness and perceived ease of use [83,84]. Unconstrained by time and space, the popularization and commercialization of optical sensors and IoT technology have boosted the use of the proposed system. Hence, technological efficacy involves individual perceptions of the system [85]. Individuals can accomplish health management through this system and attain their goals. Therefore, users with high technological efficacy can perceive more usefulness, ease of use, and less risk. Accordingly, we propose the following hypothesis:

**Hypothesis** **9-1/2/3** **(H9-1/2/3).** *TE significantly affects PU/PEU/PR.*

**Figure 2:**
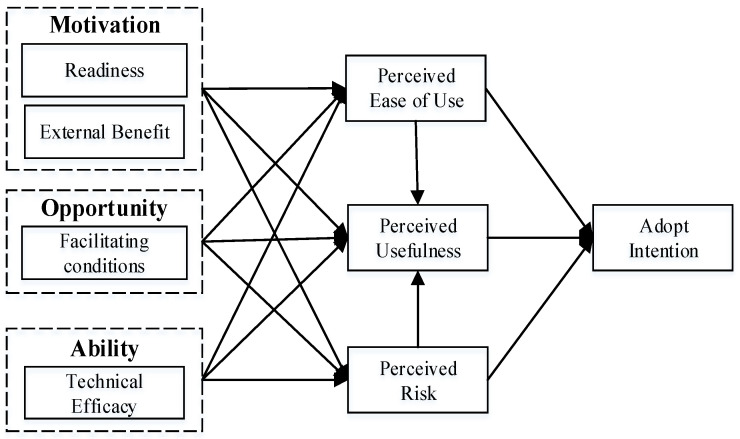
Research model.

Figure 2 depicts the research model based on the hypotheses mentioned above.

## 4. Methodology

### 4.1. Survey Design

This research used an online semi-structured questionnaire to collect raw data as this method can be done quickly and easily. That is, a smartphone/tablet with a hyperlink was used to collect raw data. The corresponding relations between latent variables are shown in Figure 2 based on the extended TAM framework, and mature scales were adopted from previous mature studies, ensuring the measures’ effectiveness. This study also modified the question items based on the initial understanding of the research context to make them more relevant to the IoT-based framework for health management. The scales for perceived usefulness and perceived ease of use were adapted from Ref. [28]. The items for adopt intention were from Ref. [86]. The items for perceived risk were from Ref. [32]. The readiness and external benefits items were from Ref. [87]. The items for perceived risk were from Ref. [76]. The scales for facilitating conditions were from Ref. [81]. The scales for technical efficacy were from Ref. [85].

It is important to note that since the scale in this research is originally in English, our study was conducted in China. Therefore, a translation and back-translation step were performed in this research to ensure that the entries corresponded to the original meaning. In order to determine the formal measurement entries, the questionnaire was presented to senior managers in the field to determine whether they thought the corresponding entries would respond to the corresponding latent variables. Based on their comments, the questionnaire was modified, and two entries were removed. All items were designed using a seven-point Likert scale, with one being “strongly disagree” and seven being “strongly agree”.

To further ensure that the subjects could understand the content of the questionnaire, 20 subjects were invited to conduct a pretest. In other words, they were asked to fill out the questionnaire and correct any awkward points in the questionnaire. The study continued to revise the questionnaire based on these subjects’ comments. Finally, the questionnaire (English version) used in this study is shown in Table 1. The responses recorded during the pilot study were subjected to Cronbach’s alpha using Stata 17.0 to measure internal reliability. All Cronbach’s alpha coefficients above 0.75 indicated that all initial scales employed were reliable measures.

### 4.2. Data Collection

The pre-prepared online questionnaire link was distributed to two different groups of respondents by invitation messages [88]. The first group comprised the elderly who do not live with their children in two cities in Anhui province of China: Wuhu and Ma’anshan. These respondents are the targets of biopharmaceutical companies as they receive regular medical checkups and are equipped with many devices with sensors to “monitor” their activities. Under the help of area managers, we distributed the questionnaire link to their clients. Before the questionnaire was filled out, an ethics statement was included, informing participants that this research was intended to be market research for a prototype product and that all questions would be used only for an academic study. If the respondent did not agree at the outset, or during or after the completion process, he or she could terminate at any time. The second group was the group of patients with chronic diseases. These respondents were contacted through agents of a health insurance company in Anhui province. We likewise included an ethics statement for this segment of participants. These subjects were also afflicted with diseases and needed to keep themselves informed about their health status. All respondents volunteered to participate in our study, and we also distributed small gifts to each participant who filled out the completed questionnaire. It should be noted that overlapping data between the first and second categories of respondents were removed from this study. In addition, it should be further noted that the research in this paper did not involve the use of an actual sensor or system, but rather a pre-test for a prototype product. Therefore, the research in this paper did not, to a certain extent, involve an invasion of individual privacy. The respondents were informed of the purpose and procedure of the study and were given feedback on the results before the study was conducted. In sum, our study was conducted as permitted by legal and ethical norms.

In order for all respondents to better understand the proposed health management system based on the combination of IoT and sensors, there was a textual description before the start of the formal questionnaire. This textual description informed the respondents that the system was a new health monitoring system based on optical sensors and other sensors and that the user’s behavioral data would be transmitted to the cloud for analysis.

This research distributed 322 questionnaires in total and received 276 valid responses. Then, a data processing program was performed to delete duplicate responses based on IP address. This research also removed questionnaires that showed a clear pattern of answers, too many of the same options, and those that took too little time to complete. Finally, only 243 questionnaires were treated as valid. Among them, 118 questionnaires were filled out by senior citizens (Group 1), and the second group of respondents filled 125 responses. Three questionnaires belonged to both the first and second groups, so the first questionnaire completed by these respondents was selected as a valid questionnaire for this research.

As shown in Table 2, the valid response rate was 70.4%. Among these respondents, 59.67% were males, and 40.33% were females. For monthly income, 38.6% were 2000 RMB or less, 25.10 were 2001–3000 RMB, and only 9.47% were above 5000 RMB. Among their ages, 15.23% were 40 years old or younger, 28.81% were 41–50 years, 7.00% were 51–60 years and 48.97% were over 60 years old. Most of the respondents were illiterate (no education, 43.62%), 19.75% had primary education, 6.17% had secondary education and 30.45% had high school education or above. Table 2 demonstrates the characteristics of the respondents.

## 5. Results

### 5.1. Descriptive Statistics

The following table depicts the data distribution characteristics of the data, including the mean, median, and standard deviation, which are shown in Table 3. The means and medians of all latent variables were between 3 and 4, and the standard deviations were within acceptable limits. This indicated that the data largely conformed to a normal distribution. In addition, the mean value of perceived risk (PR) was small (3.461), indicating that respondents perceived risk for these products at a low level.

### 5.2. Confirmatory Factor Analysis

Confirmatory factor analysis (CFA) was conducted to verify whether the relationship between a latent construct and its corresponding measure conformed to the theoretical relationship designed by the researcher [89,90]. It is a multivariate statistical technique to test the reliability and validity of many survey-based studies. In CFA, factor loading measures this matching. If a specific standardized item’s factor loading is greater than 0.70, then it represents this item attached to the latent variable well. The standardized factor loadings of eight latent variables with their respective items in this study are shown in Table 3. It can be seen that all standardized factor loadings were greater than 0.7. In addition, the goodness-of-fit (GOF) of the measurement of this study indicated that the fitness of the measurement model and data was acceptable [χ^2^/df = 1.579, CFI = 0.956, TLI = 0.947, SRMR = 0.051, RMSEA = 0.049], which indicated that the proposed model fit our data in an acceptable standard.

### 5.3. Reliability and Validity Test

As shown in Table 4, all of the composite reliability (CR) and Cronbach’s alpha coefficients were computed and found to be more than 0.80 (the recommended cut-off is 0.70). Each average variance extracted (AVE) was above 0.57 (the recommended critical value is 0.50), indicating that the measurements were reliable and passed the convergent validity test. These results indicated that the convergent validity and reliability tests had been passed.

As for discriminant validity, it reflects the distinctions between different constructs and is often assessed by the comparisons of the correlation coefficients and the square root of AVE. Discriminant validity is considered to pass if the square root of the AVE of a latent variable is greater than the correlation coefficient between the focal variable and any other variable. Taken Table 4 and Table 5 together, it can be observed that the corresponding square roots of all AVE were higher than the inter-construct correlations, confirming the accredited discriminant validity.

### 5.4. Hypothesis Testing

The structural equation model (SEM) helped explain the relationship among the mentioned latent variables defined in the model. SEM allows for the existence of interactions between variables and assumes that these effects occur simultaneously, estimating a number of equations jointly. Therefore, SEM was more suitable for this study. The fit indices [χ^2^/df = 1.540, CFI = 0.945, TLI = 0.934, SRMR = 0.054, RMSEA = 0.047] indicated that that the model was set correctly. The results are shown in Table 6.

As also reported in other TAM-based research, this study found a significant impact of perceived ease of use on perceived usefulness (β = 0.193; Z = 2.200; *p* < 0.05). Therefore, hypothesis H2 was supported. The perceived ease of use was also found to impact adopt intention (β = 0.239; Z = 3.040; *p* < 0.010), thereby confirming hypothesis H3.

Perceived usefulness had a significant impact on adopt intention (β = 0.571; Z = 9.160; *p* < 0.001), supporting hypothesis H1. Perceived risk negatively influenced adopt intention (β = −0.199; Z = −2.840; *p* < 0.010), supporting hypothesis H5. However, the perceived risk did not exert a significant impact on perceived usefulness, indicating that an individual’s perception of the system’s risk did not affect the perception of its usefulness; the two were independent of each other.

Readiness was observed to be related to perceived usefulness (β = 0.401; Z = 4.380; *p* < 0.001), supporting hypothesis H6-1. The ability factor (technique efficacy) was the only one to be related to perceived usefulness (β = 0.303; Z = 4.420; *p* < 0.001), supporting hypothesis H9-1.

Hypothesis 6-2/7-2/8-2/9-2 tests what factors influence perceived ease of use. Among them, it was found external benefits (β = 0.224; Z = 2.350; *p* < 0.050), facilitating conditions (β = 0.291; Z = 3.920; *p* < 0.001), and technique efficacy (β = 0.182; Z = 2.660; *p* < 0.010) positively related to perceived ease of use, supporting hypothesis H7-2, H8-2 and H9-2 respectively.

In addition, external benefits (β = −0.504; Z = −6.430; *p* < 0.001) and facilitating conditions (β = −0.225; Z = −3.500; *p* < 0.001) negatively influenced perceived risk; thus supporting hypothesis H7-3 and H8-3.

Thus, this study provided support for most hypotheses H1–H8, except hypotheses H4, H6-2, H6-3, H7-1, H8-1 and H9-3.

## 6. Discussion and Contributions

### 6.1. Discussion

This study aimed to discover if individuals in need of healthcare management were inclined to use the proposed IoT-based technology for health management. Combined with the TAM model and MOA theory, this study designed a comprehensive model to test the intent to adopt the technology of individuals. To be specific, readiness and external benefits reflected the individual’s inner and external motivations; facilitating conditions were the opportunity factors that facilitate use; technological efficacy stands for the ability of an individual to handle technological systems. Thus, the motivation, opportunity, and ability factors might alter individuals’ perceptions toward the proposed system.

This study further developed a prototype of the proposed system, integrating the sensors and digital technology. It provided first-hand pieces of evidence of the final system. The prototype deployed on the cloud was used to understand and translate the signals of various sensors, shared with people who were able to use it on their smartphones, so as to know their health status. Since the proposed health management system is intended to be used by average citizens, it was essential that they perceive the system as helpful, easy, and less risky as these factors are directly linked to adopt intention. This study found a positive effect of readiness and technical efficacy on perceived usefulness, indicating that such Internet-based systems are perceived to be more useful if individuals have sufficient intrinsic motivation and high levels of belief about the use of the system. However, providing external situational benefits using the IoT system can enable individuals to experience the system’s convenience, thereby improving the adopt intention in the event of an emergency, such as abnormal physical movements captured by in-house optical sensors. The opportunity and ability factors were also found to be correlated to perceived ease of use, providing pieces of evidence in offering chances to approach the proposed system. External benefits and facilitating conditions alleviated the risk perception, which indicated the importance of creating more benefits for individuals to use this system.

### 6.2. Theoretical Contributions

This study has the following theoretical contributions. First, this paper proposes an IoT-based health management system that extends the framework of sensor research. In previous sensor studies, scholars have favored the technical aspects. However, how various sensors are interpreted by individuals and adopted by those who need them has recently started to enter the research domain. The system proposed in this paper combines data signals from sensors through the cloud and big data, effectively extending the research in related fields.

Second, this study also contributes to the research literature in the field of inform systems. User acceptance of technological products has been the focus of IS research. A large number of studies have conducted in-depth research on user research on mobile health systems, i.e., the antecedents of users’ adoption of a particular mobile health application. This study proposes an integrated sensor- and mobile health-based system from a broader perspective that goes beyond user mobile health adoption studies based on a single application perspective.

Third, this paper also effectively extends the theoretical frontier of the TAM model. By organically combining the TAM model and MOA theory, this study not only reveals the impact of the TAM model on the proposed system acceptance but also further explains why individuals develop perceptions of usefulness, ease of use, and risk to the system based on their motivation, opportunity, and ability related factors.

### 6.3. Practical Implications

With the commercialization of various sensors, this study also sheds light on the psychological mechanisms that influence individuals’ intentions to use systems that combine sensors and the Internet of Things by establishing several vital variables. The system of this study can be further improved and enhanced in future practice with natural configurations of different sensors to improve individual acceptance. Furthermore, given the positive evaluation of the system by the intended users, this system could shortly be integrated and manufactured at the commercial level, with additional optical sensors to increase the capture of optical images in detecting, managing, and preventing health problems. In addition, healthcare commercial companies can benefit from this paper and design marketable systems based on IoT and medical diagnostics that are acceptable for users and benefit both users and companies.

## 7. Limitations and Further Directions

The present study also has several research limitations as well. First, the research subjects of this study were concentrated in the riverine region of eastern China, which has a higher degree of economic development, and people are more willing to accept new technologies. Future scholars could adopt a universal sample when studying related issues. Second, this study combined MOA theory and the TAM model, which extended the framework of TAM theory. Future research can investigate the problem of individuals’ technological system acceptance from other perspectives. Third, the core of the proposed IoT-based health management system lie in transforming the data from optical sensors into signals that could be understood and read. That is, the individual saw only the system, not the sensors at work. There may be cognitive differences in individuals’ attitudes towards combinations of sensors and towards individual sensors, and future research could continue to explore this in-depth.

## Figures and Tables

**Table 1:** Measure items.

Latent Variables	Items
Adopt Intention (AI)	Adopting the proposed system for my personal health management is a good idea.
	I will voluntarily use the proposed system in near future.
	I would make full use of the proposed system if I obtained it.
Perceived Ease of Use (PEU)	This proposed system would be used in a simple way.
	I think I can easily handle this proposed system with various sensors.
	The data provided by this proposed system can be understood with little effort.
Perceived Usefulness (PU)	This proposed system can improve my health status.
	This proposed system can enhance the effectiveness of detecting potential problems.
	This proposed system is needed if I face some health problems.
	This proposed system will be useful in reminding me to keep healthy.
Perceived Risk (PR)	This proposed system may run the risk of losing my losing benefits (e.g., personal information).
	This proposed system may expose my privacy.
	This proposed system may cost me time or money.
Readiness (RS)	I prefer to use the most advanced technology available.
	Technology gives me more freedom of personal management.
	Technology makes me more efficient in my life.
	I keep up with the latest technological developments in my areas of interest.
External Benefits (EB)	This proposed system represents an important value to the community.
	This proposed system attracts similar individuals like me to get benefits.
	This proposed system considerably improves the well-being of citziens.
Facilitating Condition (FC)	I was given adequate guidance to use this proposed system.
	I acquired the necessary knowledge to use this proposed system.
	This proposed system is compatible with health management.
Technical Efficacy (TE)	I am able to figure out how to use this proposed system on my own.
	I am able to figure out how to use the interface of this proposed system on my own.
	I am able to figure out how to use the different functions provided by the proposed system on my own.

**Table 2:** Descriptive statistics.

Variable	Mean	SD	Min	Median	Max
RS	4.355	1.364	1	4.333	7
EB	4.159	1.331	1.667	4	7
FC	3.683	1.248	1	3.667	7
TE	3.827	1.229	1	3.667	6.333
PEU	4.040	1.320	1	4.333	7
PU	4.486	1.249	1	4.5	7
PR	3.461	1.311	1	3	6.333
AI	4.514	1.266	1.333	4.667	7
Income	2.058	1.316	1	2	5
Education	2.235	1.291	1	2	4
Age	2.897	1.176	1	3	4
Gender	0.403	0.492	0	0	1

**Table 3:** Sample description.

Category	Value	Numbers	Percentage (%)
Gender	Male	145	59.67
	Female	98	40.33
Income (Monthly)	≤2000 RMB	116	47.74
	2001–3000 RMB	61	25.10
	3001–4000 RMB	25	10.29
	4001–5000 RMB	18	7.41
	≥5001 RMB	23	9.47
Age	≤40	37	15.23
	41–50	70	28.81
	51–60	17	7.00
	≥61	119	48.97
Education	Illiteracy	106	43.62
	Primary school or sishu	48	19.75
	Middle school	15	6.17
	High school or above	74	30.45

Note: N = 243, the same as below.

**Table 4:** Results of CFA and reliability/validity test.

Construct	Item	Factor Loading	Standard Error	Cronbach’s Alpha	CR	AVE	Sqrt (AVE)
RS	RS1	0.759	0.035	0.835	0.836	0.630	0.794
	RS2	0.820	0.030				
	RS3	0.801	0.031				
EB	EB1	0.833	0.025	0.888	0.889	0.728	0.853
	EB2	0.835	0.025				
	EB3	0.890	0.020				
FC	FC1	0.860	0.028	0.853	0.854	0.663	0.814
	FC2	0.833	0.029				
	FC3	0.744	0.035				
TE	TE1	0.737	0.047	0.806	0.811	0.589	0.767
	TE2	0.848	0.047				
	TE3	0.711	0.049				
PEU	PEU1	0.796	0.034	0.806	0.808	0.585	0.765
	PEU2	0.695	0.041				
	PEU3	0.799	0.034				
PU	PU1	0.681	0.040	0.841	0.841	0.571	0.756
	PU2	0.758	0.034				
	PU3	0.779	0.032				
	PU4	0.798	0.030				
PR	PR1	0.838	0.025	0.866	0.868	0.687	0.829
	PR2	0.863	0.023				
	PR3	0.784	0.030				
AI	AI1	0.747	0.036	0.812	0.811	0.589	0.767
	AI2	0.765	0.035				
	AI3	0.790	0.033				

**Table 5:** Correlation table.

	RS	EB	FC	TE	PEU	PU	PR	AI
RS	1							
EB	0.580 ***	1						
FC	0.200 **	0.147 *	1					
TE	0.061	0.110	0.010	1				
PEU	0.349 ***	0.314 ***	−0.0470	0.184 **	1			
PU	0.446 ***	0.346 ***	0.134 *	0.277 ***	0.401 ***	1		
PR	−0.489 ***	−0.422 ***	−0.093	−0.033	−0.525 ***	−0.334 ***	1	
AI	0.398 ***	0.321 ***	0.140 *	0.160 *	0.342 ***	0.646 ***	−0.293 ***	1

Note: *** *p* < 0.001, ** *p* < 0.01, * *p* < 0.05, the same as below.

**Table 6:** Hypotheses testing results.

Hypothesis	Relationship	Estimate	S.E.	Z	*p*	95% CI		Result
H1	PU→AI	0.571	0.062	9.160	0.000	0.449	0.693	Supported
H2	PEU→PU	0.193	0.088	2.200	0.028	0.021	0.365	Supported
H3	PEU→AI	0.239	0.079	3.040	0.002	0.085	0.394	Supported
H4	PR→PU	0.011	0.104	0.110	0.914	−0.193	0.216	Not Supported
H5	PR→AI	−0.199	0.070	−2.840	0.004	−0.336	−0.062	Supported
H6-1	RS→PU	0.401	0.092	4.380	0.000	0.222	0.580	Supported
H6-2	RS→PEU	0.159	0.101	1.580	0.113	−0.038	0.357	Not Supported
H6-3	RS→PR	−0.159	0.086	−1.850	0.064	−0.327	0.009	Not Supported
H7-1	EB→PU	−0.029	0.105	−0.270	0.784	−0.234	0.177	Not Supported
H7-2	EB→PEU	0.224	0.095	2.350	0.019	0.037	0.411	Supported
H7-3	EB→PR	−0.504	0.078	−6.430	0.000	−0.657	−0.350	Supported
H8-1	FC→PU	0.111	0.077	1.430	0.152	−0.041	0.262	Not Supported
H8-2	FC→PEU	0.291	0.074	3.920	0.000	0.145	0.436	Supported
H8-3	FC→PR	−0.225	0.064	−3.500	0.000	−0.352	−0.099	Supported
H9-1	TE→PU	0.303	0.069	4.420	0.000	0.169	0.438	Supported
H9-2	TE→PEU	0.182	0.068	2.660	0.008	0.048	0.316	Supported
H9-3	TE→PR	0.053	0.059	0.900	0.370	−0.062	0.168	Not Supported

## Data Availability

Not Applicable.
